# Evaluation of a Urine Pooling Strategy for the Rapid and Cost-Efficient Prevalence Classification of Schistosomiasis

**DOI:** 10.1371/journal.pntd.0004894

**Published:** 2016-08-09

**Authors:** Nathan C. Lo, Jean T. Coulibaly, Eran Bendavid, Eliézer K. N’Goran, Jürg Utzinger, Jennifer Keiser, Isaac I. Bogoch, Jason R. Andrews

**Affiliations:** 1 Division of Infectious Diseases and Geographic Medicine, Stanford University School of Medicine, Stanford, California, United States of America; 2 Unité de Formation et de Recherche Biosciences, Université Félix Houphouët-Boigny, Abidjan, Côte d’Ivoire; 3 Centre Suisse de Recherches Scientifiques en Côte d’Ivoire, Abidjan, Côte d’Ivoire; 4 Swiss Tropical and Public Health Institute, Basel, Switzerland; 5 University of Basel, Basel, Switzerland; 6 Division of General Medical Disciplines, Stanford University, Stanford, California, United States of America; 7 Center for Health Policy and the Center for Primary Care and Outcomes Research, Stanford University, Stanford, California, United States of America; 8 Department of Medicine, University of Toronto, Toronto, Canada; 9 Divisions of Internal Medicine and Infectious Diseases, Toronto General Hospital, University Health Network, Toronto, Canada; University of Iowa College of Public Health, UNITED STATES

## Abstract

**Background:**

A key epidemiologic feature of schistosomiasis is its focal distribution, which has important implications for the spatial targeting of preventive chemotherapy programs. We evaluated the diagnostic accuracy of a urine pooling strategy using a point-of-care circulating cathodic antigen (POC-CCA) cassette test for detection of *Schistosoma mansoni*, and employed simulation modeling to test the classification accuracy and efficiency of this strategy in determining where preventive chemotherapy is needed in low-endemicity settings.

**Methodology:**

We performed a cross-sectional study involving 114 children aged 6–15 years in six neighborhoods in Azaguié Ahoua, south Côte d’Ivoire to characterize the sensitivity and specificity of the POC-CCA cassette test with urine samples that were tested individually and in pools of 4, 8, and 12. We used a Bayesian latent class model to estimate test characteristics for individual POC-CCA and quadruplicate Kato-Katz thick smears on stool samples. We then developed a microsimulation model and used lot quality assurance sampling to test the performance, number of tests, and total cost per school for each pooled testing strategy to predict the binary need for school-based preventive chemotherapy using a 10% prevalence threshold for treatment.

**Principal Findings:**

The sensitivity of the urine pooling strategy for *S*. *mansoni* diagnosis using pool sizes of 4, 8, and 12 was 85.9%, 79.5%, and 65.4%, respectively, when POC-CCA trace results were considered positive, and 61.5%, 47.4%, and 30.8% when POC-CCA trace results were considered negative. The modeled specificity ranged from 94.0–97.7% for the urine pooling strategies (when POC-CCA trace results were considered negative). The urine pooling strategy, regardless of the pool size, gave comparable and often superior classification performance to stool microscopy for the same number of tests. The urine pooling strategy with a pool size of 4 reduced the number of tests and total cost compared to classical stool microscopy.

**Conclusions/Significance:**

This study introduces a method for rapid and efficient *S*. *mansoni* prevalence estimation through examining pooled urine samples with POC-CCA as an alternative to widely used stool microscopy.

## Author Summary

Schistosomiasis is a disease caused by parasitic worms that affects over 250 million people. The global control strategy is regular deworming of school-aged children. Before deworming campaigns can be conducted, one must know where the disease is present. The current method requires collection of individual stool and urine samples that are examined under a microscope by trained laboratory technicians. We present an alternative method that can inform where schistosomiasis is present in above 10% of the population, which is the threshold at which school-based deworming is recommended. The proposed strategy involves pooling multiple urine samples and using a rapid diagnostic test. The goal is to reduce the number of tests, cost, time, and laboratory infrastructure to guide decision-making. We collected data in Côte d’Ivoire to evaluate this new diagnostic procedure of pooling urine, and used computer simulation to predict its performance in classification of communities above or below the 10% threshold. We found that the urine pooling strategy with a pool size of 4 reduced the number of tests and cost compared to the current standard method, while maintaining the same accuracy. Our findings suggest that this strategy may be an effective and cost-saving method compared to traditional microscopy.

## Introduction

Schistosomiasis is a disease caused by parasitic worms of the genus *Schistosoma*, and affects over 250 million people residing in the world’s poorest regions [1]. Historically, treatment programs have focused on control of disease morbidity [2–4]. However, the focus of these programs has recently shifted toward a goal of interrupting disease transmission and local elimination of helminth infections [4]. The World Health Organization (WHO) recommends a strategy of preventive chemotherapy (often known as ‘mass drug administration’) for control and elimination of schistosomiasis [2, 3]. The preventive chemotherapy program provides widespread empiric treatment with praziquantel, and traditionally focuses upon school-aged children [2, 3]. The frequency of preventive chemotherapy, if necessary, is based upon the infection prevalence [2, 3].

Most schistosomiasis control programs utilize prevalence estimates from survey samples of selected schools to guide treatment decisions for preventive chemotherapy [2, 3]. The WHO currently recommends surveying one sentinel site (i.e., at least 50 children in one school) per 200,000–300,000 children in a homogeneous “ecological zone” [3]. For each child in a sentinel site, a fecal and urine sample is obtained. The stool is prepared using the Kato-Katz thick smear method and examined under a microscope (referred to as traditional stool microscopy) for the eggs of intestinal schistosomiasis (caused by *Schistosoma mansoni* and *S*. *japonicum*). The urine is tested for microhematuria or filtered and examined under a light microscope for the eggs of *S*. *haematobium*. These strategies are the current standard for prevalence estimation, but require laboratory infrastructure, trained personnel for slide preparation and interpretation, lack adequate sensitivity to detect low intensity infections, and are time- and resource-intensive [3, 5, 6].

These barriers have contributed to the challenge of mapping the prevalence of schistosomiasis in endemic countries, which is necessary to inform where preventive chemotherapy should be implemented. Furthermore, a high-resolution understanding of local prevalence is especially important for schistosomiasis compared to other helminth infections, because schistosomiasis is highly geographically focal due to dependence on freshwater for completion of the life cycle [7].

The pooling of urine samples with the point-of-care circulating cathodic antigen (POC-CCA) cassette test may provide a cost-efficient alternative to traditional stool microscopy. The pooling of biological specimens (including feces and urine) as an efficient methodology for disease screening is common across veterinary parasitology, HIV, and other diseases [8, 9]. The POC-CCA test is a rapid diagnostic test that uses urine for the binary detection of *S*. *mansoni*. Most notably, the POC-CCA test retains high sensitivity at low intensity infections and does not read positive once an infection is resolved making it suitable for application in pooled sampling [5, 10–12]. However, there are currently no studies that have examined the diagnostic characteristics, optimal pooling size, or cost-efficiencies associated with a urine pooling methodology using the POC-CCA cassette test. Furthermore, since the preventive chemotherapy strategy is based upon a broad prevalence categorization (WHO groups settings by <10%, 10–50%, or >50% prevalence) rather than a specific prevalence value, a simplified classification tool can be employed. Lot quality assurance sampling (LQAS) is an approach to evaluate the accuracy of classification of unknown entities into binary or multiple groups, according to pre-defined thresholds [13–15]. This tool has been well characterized in the helminthiasis literature [13–15], and provides an attractive option for estimating the broad prevalence category to reduce number of tests and cost.

To address this critical need, we conducted an empirical evaluation of the accuracy of urine pooling with the POC-CCA cassette test, and then applied simulation modeling using a LQAS framework to evaluate classification accuracy and efficiency for informing targeted treatment of schistosomiasis.

## Methods

### Ethics Statement

This study was approved by the Institutional Review Board for Human Subjects Research at Stanford University School of Medicine (Stanford, CA, United States of America) and ethical clearance was obtained from the Ministry of Health and Public Hygiene of Côte d’Ivoire (CNER, reference no. 037/MSLS/CNER-dkn). We obtained written informed consent from parents/guardians and oral assent from children in Azaguié Ahoua, Côte d’Ivoire. All data were coded and treated as confidential personal health information. After completion of the study, all study participants received praziquantel (single 40 mg/kg oral dose) and albendazole (single 400 mg oral dose) at no cost as per national guidelines [2, 3].

### Study Setting and Population

This cross-sectional study was performed in August 2015 at schools from six neighborhoods in the Azaguié health district (geographic coordinates: 5° 37' 40" N latitude and 4° 5' 12" W longitude) of Côte d’Ivoire, located at 40 km north from Abidjan. These settings ranged from moderate to high endemicity for *S*. *mansoni* to ensure a sufficient number of positive urine and stool samples were obtained. We selected 180 children between the ages of 6 and 15 years, informed by sample size estimations for diagnostic tests (S1 Text) [16].

### Field and Laboratory Procedures

A detailed census was carried out in early August 2015 to determine the number of school-aged children per neighborhood in Azaguié Ahoua village. Based on that list, 30 children were randomly selected per neighborhood. The purpose and procedures of the study were explained to the village and health authorities. For children who provided oral assent, and whose parent/guardian provided written informed consent, we obtained two stool and two midstream urine samples over two days. Urine was collected between 10:00 and 12:00 hours.

For both stool sample collected over consecutive days, duplicate Kato-Katz thick smears were prepared the same day as collection following standard protocol, for a total of four Kato-Katz thick smears per child [17]. Slides were labeled with a de-identified code and read by experienced laboratory technicians using light microscopy. The presence and quantity of helminth eggs was counted on each slide for *S*. *mansoni*. For quality control purposes, we randomly selected 10% of the Kato-Katz thick smears, including both positive and negative slides, for re-examination by a senior technician. If discrepancies above the tolerance margin were noted, the results were discussed with the technicians and the slides were read a third time to reach agreement (S1 Text).

All of the first day urine samples were tested using the POC-CCA cassette test (Rapid Medical Diagnostics; Pretoria, South Africa, batch #50174) on the same day of sample collection. To perform the test, one drop of urine was placed into the POC-CCA cassette well followed by one drop of the test buffer. Two blinded study personnel experienced with POC-CCA read the tests independently 20 min after the addition of buffer. In cases of disagreement, a third blinded technician read the results and a decision was made based on agreement of at least two out of three individuals. Tests were read as negative, trace positive, 1+, 2+, or 3+ according to the color intensity of the test band, and tests that did not develop the control band were repeated.

For the pooling of urine samples, we first identified individuals as positive or negative using quadruplicate Kato-Katz thick smears and a single POC-CCA test. Positive samples were from children with a positive Kato-Katz and POC-CCA test, while negative samples were from children with a negative Kato-Katz and POC-CCA test. Hence, we only included samples with concordant results from both tests. One positive urine sample (~5 ml) was then combined with equal volumes of three (n = 4), seven (n = 8), or 11 (n = 12) negative urine samples. All samples were poured into a urine collection container, and a brief mixing step was done with the disposable pipette provided with the POC-CCA test kit. We then performed the POC-CCA test as described above.

### Statistical Analysis

We calculated the sensitivity and specificity of quadruplicate Kato-Katz thick smears and the individual POC-CCA test using latent class analysis [18]. This analytical strategy combines prior knowledge on sensitivity and specificity and the observed data to simultaneously calculate point estimates and 95% credible intervals around the sensitivity and specificity for two or more diagnostics tests without assuming any test as the ‘gold’ standard [18]. We used the Gibbs sampler model for two diagnostic tests with assumption of independence, and derived a prior distribution following consensus from literature using a beta distribution (S1 Text) [5, 10, 12, 18, 19]. Only children with complete data records (i.e., quadruplicate Kato-Katz thick smears and POC-CCA test) were included in the final analysis. We calculated eggs per gram of feces (EPG) from the four Kato-Katz thick smears using an arithmetic mean and the conventional 24-fold multiplier [20]. Sensitivity of the pooled samples was calculated assuming that a single positive urine sample classified the entire pool as positive. We used a logistic regression to model the sensitivity of pooled urine samples (with pool sizes of 4, 8, and 12) as a function of the EPG of the one infected urine. For each individual, we defined the independent variable as the arithmetic mean EPG from quadruplicate Kato-Katz thick smears and the dependent binary outcome as whether or not the pooled urine was read as positive with the POC-CCA cassette test. All analyses were conducted by considering the POC-CCA trace result as positive (POC-CCA(tr+)) and negative (POC-CCA(tr-)) to examine the impact of this interpretation on results [2, 3].

### Model and Assumptions

We developed an individual-level stochastic decision analytic model (first order Monte Carlo simulation; microsimulation) for diagnosis of schistosomiasis to test the performance of the urine pooling strategies using the LQAS classification tool. We compared five strategies: (i) duplicate Kato-Katz thick smears (WHO standard); (ii) single POC-CCA test; (iii) pooled POC-CCA test (n = 4); (iv) pooled POC-CCA test (n = 8); and (v) pooled POC-CCA test (n = 12). A simulated cohort of 100,000 individuals was created for each prevalence value (0–20%), with assignment of infection status and EPG (when applicable) to each person. We assumed a negative binomial statistical distribution, and used a specified prevalence and inferred infection intensity to simulate the distribution of egg counts in the cohort (S1 Text) [21].

In the microsimulation, a sample of individuals was randomly selected from the simulated cohort. Each individual was assigned to one of four mutually exclusive states (true positive, true negative, false positive, or false negative) using the computed sensitivity and specificity of the respective diagnostic strategy and knowledge of an individual’s “true” infection status (from simulated egg counts). The sensitivity (in relation to infection intensity) and specificity for duplicate Kato-Katz thick smears for individual samples was derived from literature [5], while we used the sensitivity (irrespective of infection intensity) and specificity from the latent class analysis for single POC-CCA. For pooled POC-CCA test strategies with more than one positive sample, we conservatively used the total EPG count to relate the infection intensity to our pooling data (in which only one urine was infected) and computed sensitivity using the logistic model (S1 Text; Fig A1–A2 in S1 Text). To address the impact of urine pooling on specificity, we used the POC-CCA specificity estimate from the Bayesian latent class analysis (which assumes no diagnostic ‘gold’ standard), and accounted for the increased probability of including a false positive by adding more urine samples that could be false positives. We assumed the effect of diluting a false positive urine sample was comparable to diluting a light-intensity infected urine sample (S1 Text). Ultimately, the dilution effect counteracted the potential for decreased specificity from sample pooling (S1 Text).

As preventive chemotherapy strategies are based on classifying regions into a prevalence bin (i.e., above or below a predefined prevalence threshold), we chose our primary outcome as the probability of correct binary classification around one prevalence threshold. Specifically, we tested the WHO-recommended prevalence threshold for preventive chemotherapy against schistosomiasis (10% prevalence) [2, 3]. We defined classification certainty as the proportion of correctly categorized settings within 5% of the prevalence threshold, following estimates from prior studies [14]. The decision rule was chosen based on the median number of positive tests from the microsimulation at the prevalence (10%; S1 Text). The microsimulation was run 10,000 times for each strategy using a range of 15 to 500 tests for each strategy. We estimated the number of correct categorical classifications for each strategy at all prevalences. We also tested the potential to reduce the number of tests with the urine pooling strategies compared to stool microscopy, while maintaining the same level of classification accuracy. We compared our results against previous analytical approaches to LQAS [14], which did not account for imperfect test accuracy. A loess algorithm was applied for visualization and interpolation.

The cost of each strategy was estimated from recent literature, incorporating costs for supplies, labor, and pooling. We added an additional US$ 0.50 per extra sample in the pooling strategy to account for personnel time and collection container costs (S1 Text) [6]. The total cost for POC-CCA pool sizes of 4, 8, and 12 urines was estimated at US$ 6.63, US$ 8.63, and US$ 10.63, respectively; Kato-Katz was estimated at US$ 3.99 (S1 Text).

Data were recorded in a Microsoft Excel spreadsheet, and statistical analysis and data visualization were performed with Python and R 3.1.1 (R Foundation for Statistical Computing; Vienna, Austria). The authors support the importance of data sharing and transparency in research; hence, full model code and data are available upon request to the corresponding author.

### Sensitivity Analysis

We performed a series of sensitivity analyses to assess the robustness of our findings. We conducted one-way sensitivity analyses to examine the effect of individual parameters on the total cost of the urine pooling strategy for a 90% level of certainty in classification. We varied number of tests, cost estimates, sensitivity, specificity, and the dilution effect on light infections. We also tested the robustness of results against setting-specific epidemiologic differences, where the EPG distribution was varied for the same prevalence. We repeated the main analysis using a lower proposed prevalence threshold based on recent cost-effectiveness modeling (5% prevalence instead of the current 10% WHO cutoff) [21, 22].

## Results

### Sensitivity and Specificity of Individual and Pooled Diagnostic Strategies

From the 114 school-aged children with complete data, 59.6% were positive by quadruplicate Kato-Katz thick smears, 69.3% were positive by single POC-CCA(tr+), and 50.0% were positive by single POC-CCA(tr-) (Table 1). Using a latent class model, we estimated the sensitivity and specificity of quadruplicate Kato-Katz thick smears at 78.2% (95% CI: 71.0–84.5%) and 96.1% (95% CI: 90.8–98.8%), respectively (Table 2). We estimated the sensitivity of POC-CCA(tr+) test at 95.1% (95% CI: 89.2–98.5%) and POC-CCA(tr-) test at 74.4% (95% CI: 63.8–83.7%). The specificity of POC-CCA(tr+) test was 82.9% (95% CI: 71.5–91.6%) and POC-CCA(tr-) test was 94.5% (95% CI: 86.9–98.5%).

**Table 1:** Demographic and epidemiologic characteristics of six neighborhoods in Azaguié Ahoua, south Côte d’Ivoire.

	Setting A	Setting B	Setting C	Setting D	Setting E	Setting F	Overall
Number of children	20	12	16	22	21	23	114
Kato-Katz^a^							
Number positive	14	7	9	17	10	11	68
Mean EPG^c^	174	199	631	337	439	214	323
POC-CCA^b^							
Number positive (trace positive)	2	3	2	2	10	3	22
Number positive (1+ or greater)	14	5	9	15	3	11	57
Number negative	4	4	5	5	8	9	35

^a^Data from quadruplicate Kato-Katz thick smears

^b^Data from single POC-CCA urine cassette test

^c^Arithmetic mean EPG calculated from infected individuals.

**Table 2:** Sensitivity and specificity of Kato-Katz and the POC-CCA test using a latent class model.

	Kato-Katz (quadruplicate)	POC-CCA test
***POC-CCA(tr+)***		
Sensitivity	78.2 (95% CI: 71.0–84.5)	95.1 (95% CI: 89.2–98.5)
Specificity	96.1 (95% CI: 90.8–98.8)	82.9 (95% CI: 71.5–91.6)
***POC-CCA(tr-)***		
Sensitivity	78.9 (95% CI: 71.5–85.4)	74.4 (95% CI: 63.8–83.7)
Specificity	95.4 (95% CI: 89.2–98.6)	94.5 (95% CI: 86.9–98.5)

Note: For microsimulation, Kato-Katz sensitivity was estimated from literature as a function of infection intensity (EPG)

The overall sensitivity of the urine pooling strategy (POC-CCA(tr+)) was 85.9%, 79.5%, and 65.4% for pool sizes of 4, 8, and 12 (Table 3). For the urine pooling strategy (POC-CCA(tr-)), the sensitivity was 61.5%, 47.4%, and 30.8% for pool sizes of 4, 8, and 12. The modeled specificity ranged from 94.0–97.7% for the urine pooling strategies. The sensitivity of each strategy increased for detection of moderate and heavy infections.

**Table 3:** Sensitivity and specificity of the POC-CCA test with a urine pooling strategy.

Test characteristic	Category	Number	Dilution, n = 4	Dilution, n = 8	Dilution, n = 12
**Sensitivity**	Overall	78	85.9	79.5	65.4
*POC-CCA(tr+)*	Light infection	43	76.7	69.8	48.8
	Moderate infection	16	93.8	93.8	87.5
	Heavy infection	19	100.0	89.5	84.2
**Sensitivity**	Overall	78	61.5	47.4	30.8
*POC-CCA(tr-)*	Light infection	43	41.9	30.2	4.6
	Moderate infection	16	81.3	68.8	62.5
	Heavy infection	19	89.5	68.4	63.2
**Specificity**					
*POC-CCA(tr+)*	Tested^a^	10	90.0	100.0	100.0
	Model conditions^b^	-	77.2	75.9	78.6
**Specificity**					
*POC-CCA(tr-)*	Tested^a^	10	100.0	100.0	100.0
	Model conditions^b^	-	94.0	94.9	97.7

^a^Tested specificity was calculated using urine pools that were each negative by single POC-CCA test (see S1 Text).

^b^Model conditions specificity was calculated using the specificity of single POC-CCA with the assumption that the dilution effect upon false positives would mimic very light intensity infections (see Methods and S1 Text).

**Fig 1:**
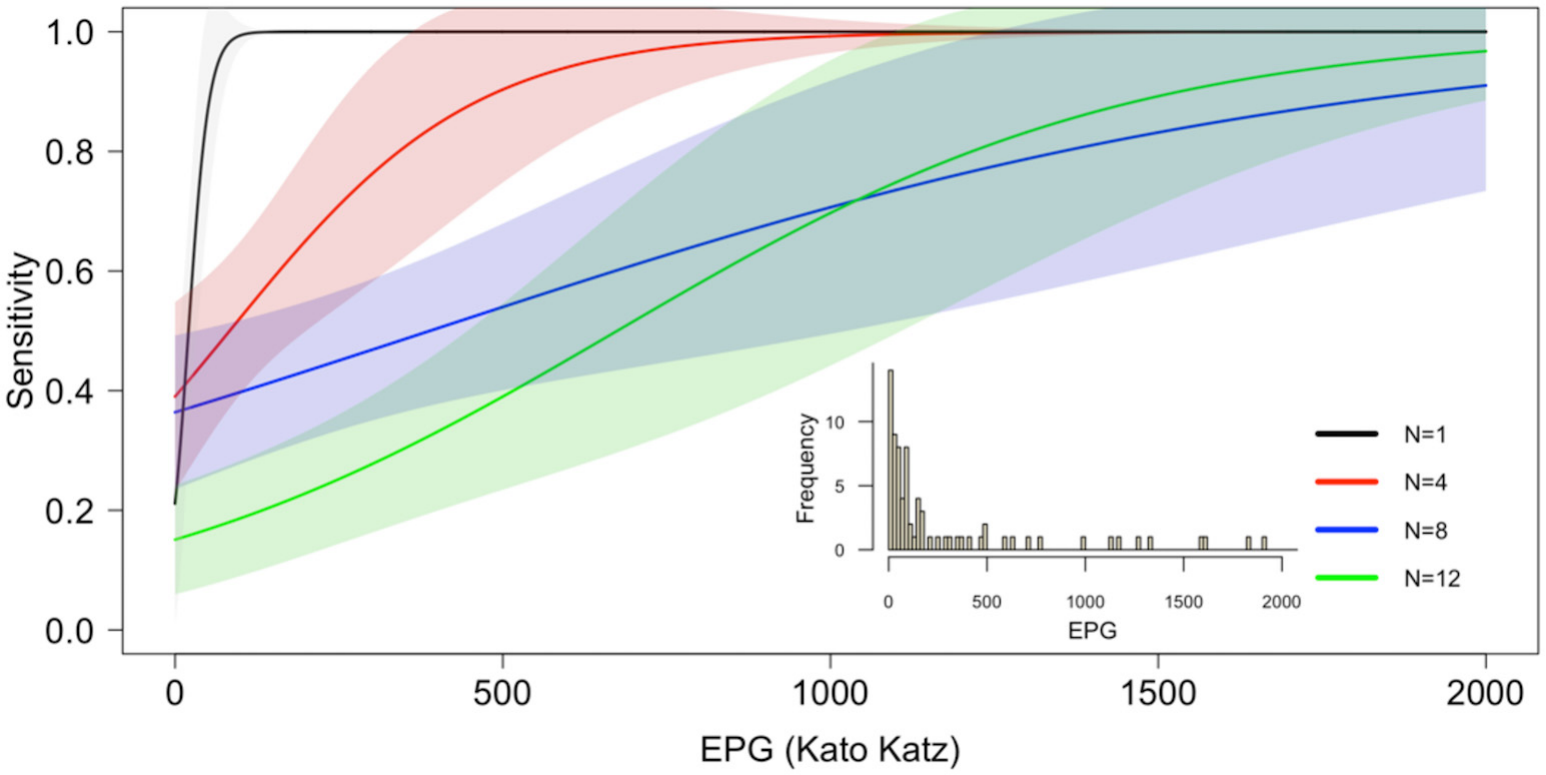
Modeled estimation of the sensitivity of the urine pooling strategy. Positive urine sample confirmed by quadruplicate Kato-Katz thick smears and single POC-CCA urine cassette test were combined with three (n = 4), seven (n = 8), or 11 (n = 12) negative urine samples. A logistic regression was used to model sensitivity of the pooled urine samples (n = 4, 8, and 12) as a function of the infection intensity (EPG) of the one infected urine. The distribution of *S*. *mansoni* infections used in this model included light, moderate, and heavy intensity infections (figure inset).

The sensitivity of each pooled strategy (POC-CCA(tr-)) was modeled as a logistic function with respect to infection intensity, measured in EPG (Fig 1). Sensitivity was strongly positively correlated with EPG and was negatively associated with larger pool size, particularly at lower infection intensities.

### Performance of the Urine Pooling Strategy and Traditional Stool Microscopy in Prevalence Classification Using the Microsimulation Model

**Fig 2:**
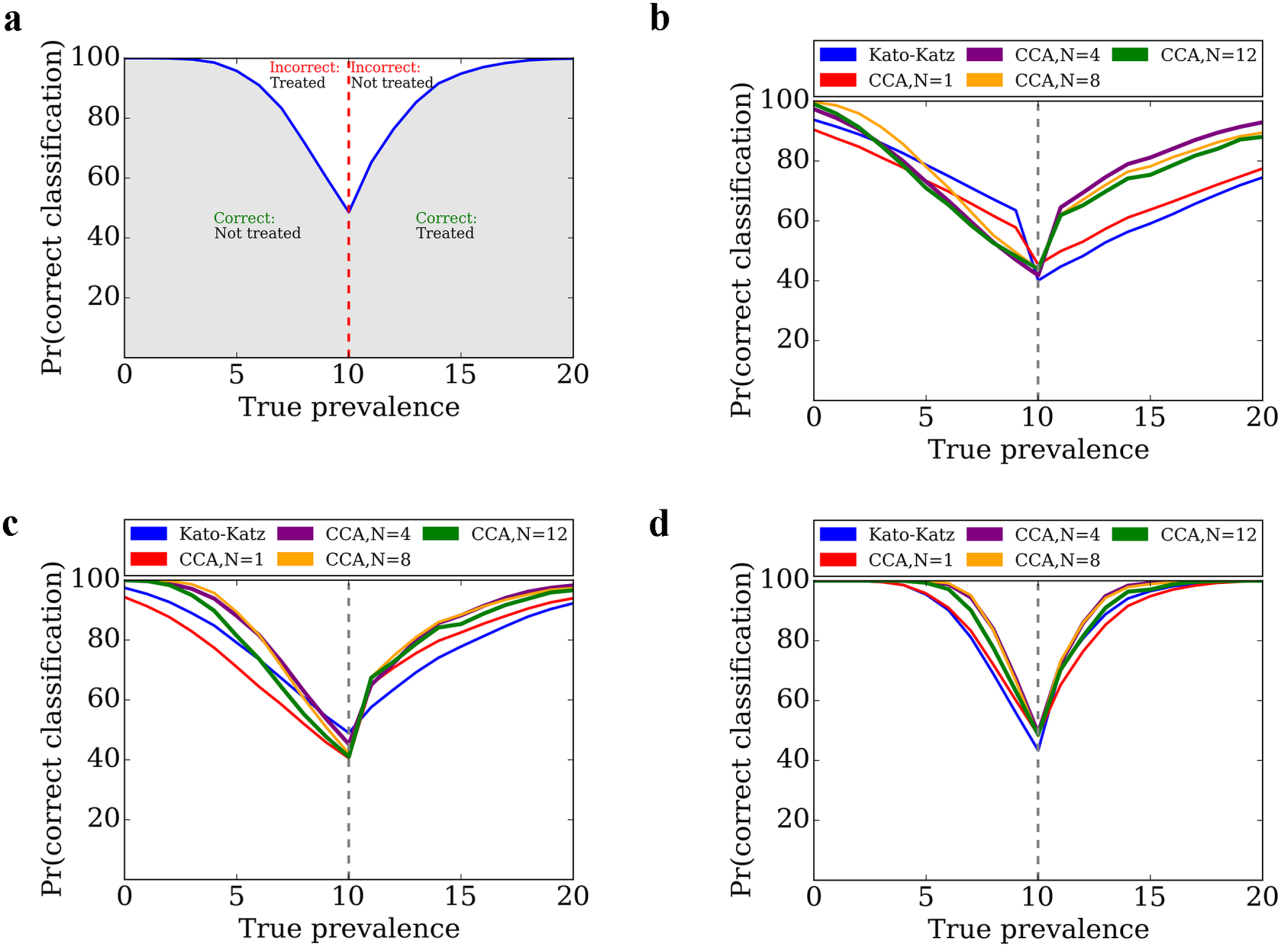
Operating characteristic curves for microsimulation analysis of urine pooling strategy to rapidly map the prevalence of schistosomiasis and inform preventive chemotherapy. Using primary data on urine pooling strategies (n = 4, 8, and 12 samples), we used a microsimulation to model the proportion (Pr) of correct binary classification around a prevalence threshold to indicate need for school-based preventive chemotherapy according to WHO (10% prevalence). The interpretation of the curves is provided in (a). The results are presented for: (b) 20 tests; (c) 50 tests; and (d) 250 tests. We compared traditional stool microscopy (duplicate Kato-Katz thick smears derived from one stool sample), single POC-CCA test, and the three urine pooling strategies (pool sizes of 4, 8, and 12) using the WHO 10% prevalence threshold. The strategies performed well within the center of a prevalence categorization, but poorly at the boundary between two prevalence categories. Increased number of tests resulted in lower classification error.

**Fig 3:**
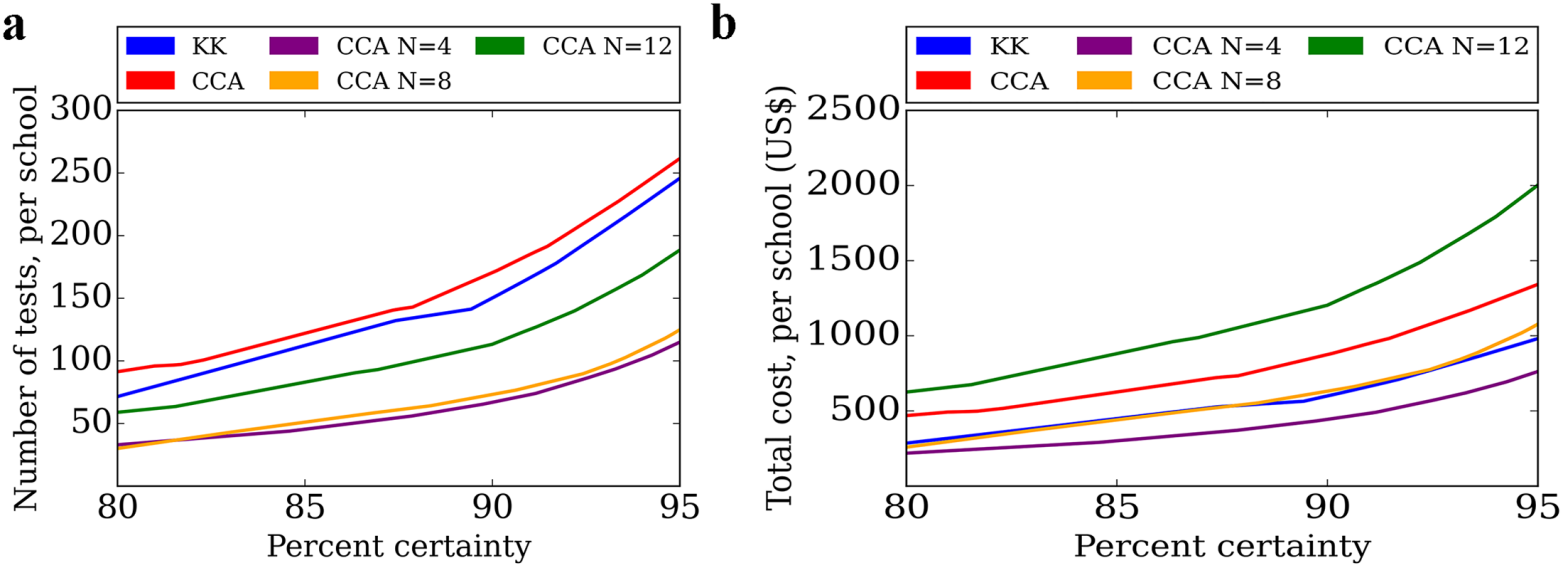
Number of tests and total costs per school from microsimulation analysis of urine pooling strategy and traditional stool microscopy. The urine pooling strategy (n = 4, 8, and 12 pool) was compared against stool microscopy to estimate (a) the total number of tests per school and (b) total cost per school for identical level of certainty in binary classification on need for preventive chemotherapy. We used a 10% prevalence threshold in this base case analysis. A loess algorithm was applied for visualization.

We used a microsimulation to evaluate the diagnostic performance of three urine pooling strategies (n = 4, 8, and 12 pooled samples) to give a binary prediction for informing preventive chemotherapy programs in simulated cohorts across a prevalence range from 0 to 20%. The three pooling strategies gave comparable, and often superior, classification performance to traditional stool microscopy for the same number of tests (Figs 2 and 3). As expected, classification error for all strategies was highest when the true prevalence was near the prevalence threshold, and classification improved further away from the prevalence threshold. For 80% and 90% certainty of correct classification of communities (± 5% around the 10% prevalence threshold), traditional stool microscopy required 71 and 150 tests, while urine pooling (n = 4) needed 33 and 67 tests, and urine pooling (n = 8) needed 29 and 73 tests, respectively.

### Total Cost and Number of Tests with Urine Pooling Strategy

The urine pooling strategies (n = 4, 8, and 12) reduced the number of tests, while achieving the same accuracy as traditional stool microscopy across the full range of certainties in classification (Fig 3). Only the pooling strategy (n = 4) demonstrated cost savings compared to traditional stool microscopy. The pooling strategies (n = 8 and 12) did not reduce the total cost. This result remained robust when evaluating the number of tests and total cost per correctly classified school (S1 Text).

### Results from Sensitivity Analysis

**Fig 4:**
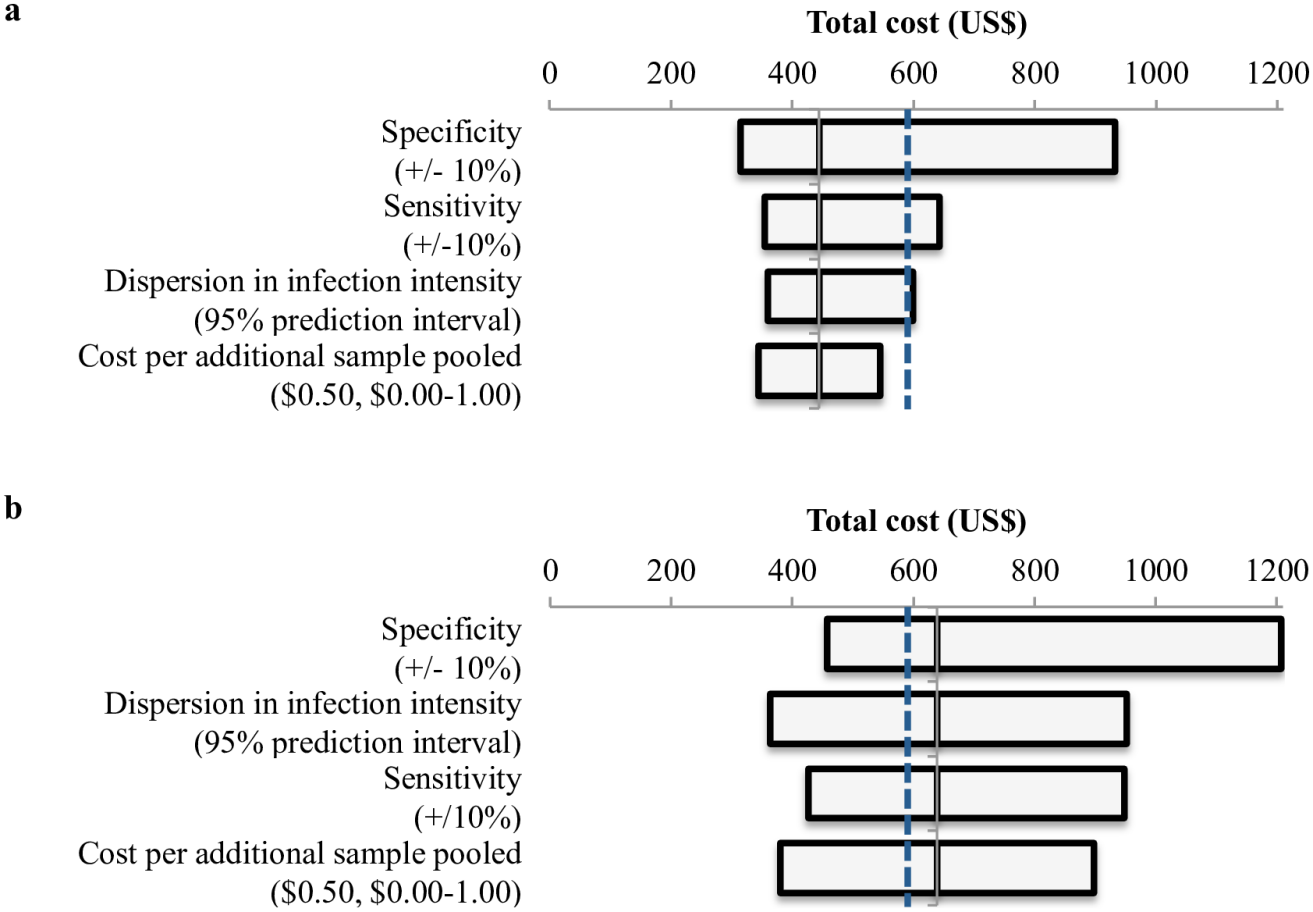
One-way sensitivity analysis of microsimulation. This analysis tested the effect of changing individual model parameters on the total cost of the urine pooling strategy for (a) pool of 4 and (b) pool of 8. The horizontal bar represents the total cost to achieve 90% level of certainty (±5% around the prevalence threshold) in classification across a range of values for the tested parameter. The y-axis (solid black line) represents the total cost of the urine pooling strategy under base case assumptions. The vertical dashed blue line represents the total cost of traditional stool microscopy under base case assumptions, and all strategies to the left of this line indicate a cost saving advantage of the urine pooling strategy.

The one-way sensitivity analyses found that our primary finding–urine pooling strategy (n = 4) yielded cost savings when compared to stool microscopy–was robust in the majority of alternative assumption on pooling cost, setting-specific epidemiologic differences, sensitivity, and specificity except on the upper ranges (Fig 4). Under some assumptions, pooling 8 samples also yielded cost savings. The overall study findings were comparable when using a prevalence threshold of 5% (S1 Text).

## Discussion

This study found that a urine pooling strategy using the commercialized POC-CCA test could be more efficient than individual-based surveys with traditional stool microscopy in informing where preventive chemotherapy against schistosomiasis is needed in low-endemicity settings. We characterized the sensitivity and specificity of urine pooling with the POC-CCA test across multiple dilutions, and used a statistical classification tool (lot quality assurance sampling) to operationalize this pooling strategy as a binary predictor of whether or not preventive chemotherapy is needed in low-endemicity settings. While sensitivity declined, as expected, at higher pool sizes, this loss was offset by the efficiency gains in screening multiple samples simultaneously. Through simulation modeling, we found that the pooling strategy reduced the number of tests and total cost, while achieving the same performance as traditional stool microscopy. These findings support the need for further validation of the urine pooling strategy in low-endemicity and near-elimination settings as a rapid, cost-saving alternative to traditional stool microscopy.

As the global strategy shifts from morbidity control to a goal of disease elimination and treatment programs are expanded, high-resolution mapping of where schistosomiasis is prevalent is crucial, especially since this disease is highly geographically focal [4, 23]. Additionally, some settings will reduce their prevalence to below the 10% threshold in the school-aged child population, and hence, preventive chemotherapy would no longer be indicated. Once settings do approach elimination, rigorous monitoring and surveillance will be essential to detect disease rebound. For all these reasons, a rapid and inexpensive approach to inform settings on the need for preventive chemotherapy will be crucial.

We evaluated the diagnostic performance and cost of a pooled urine methodology with multiple pool sizes (n = 4, 8, and 12). The use of sample pooling for diagnostic screening of parasitic and other infectious diseases has been previously demonstrated [8, 9]. We found that the urine pool size of 4 performed optimally with a balance of good sensitivity and specificity and relatively low cost. Across a range of certainties in classification, the urine pooling strategy (n = 4) reduced the number of tests and total cost with the same performance as traditional stool microscopy. The pool sizes of 8 and 12 had lower sensitivity, and ultimately offered limited test or cost savings except under select conditions. In this analysis, we treated POC-CCA trace positive test results as negative, which resulted in lower sensitivity but higher specificity. While treating trace positive results as positive greatly improved sensitivity, this resulted in substantially more false positives, which was compounded by the effect of sample pooling. We also evaluated single POC-CCA with the latent class model, which does not assume any diagnostic ‘gold’ standard test, and found our results for sensitivity and specificity in broad agreement with previous studies [5, 12, 19, 24]. Notably, our POC-CCA specificity estimate was lower than previous estimates making our modeling results conservative [5, 12, 24].

The LQAS classification tool–which is used for binary categorization based on a pre-defined threshold–is a useful method to reduce sampling effort and maintain accuracy [13, 14]. Our study focused on providing a binary classification around the 10% prevalence threshold (within ±5% prevalence) for schistosomiasis, which is the threshold that school-based preventive chemotherapy is recommended. We did not assess multiple category-LQAS since the pooling strategy is best suited for binary classification, and the 10% threshold allowed for identification of settings where treatment would be indicated. Rigorous study has been given to the application of LQAS in classification of helminth prevalence [13, 14], although previous studies have assumed perfect sensitivity and specificity and estimated a sample size of 15 tests for 80% certainty (± 6.5% around the 10% prevalence threshold). Under these conditions, our microsimulation corroborated the estimated sample size. However, when we incorporated imperfect diagnostics, we found that an increased sample size was needed. For 80% and 90% certainty, traditional stool microscopy required 71 and 150 tests, while urine pooling (n = 4) needed 33 and 67 tests. This suggests the importance of accounting for imperfect test characteristics and random sampling in LQAS calculations, and the utility of urine pooling to decrease the number of necessary tests. Notably, sampling effort for both traditional stool microscopy and urine pooling with the LQAS tool is still lower than current WHO recommendations that suggest 250–500 school-aged children (50 per school) [2, 3, 14]. This demonstrates the value of LQAS to decrease sampling effort, total cost, and time necessary to correctly classify a setting.

We evaluated a sample pooling strategy with the POC-CCA urine cassette tests, which is designed to detect *S*. *mansoni* and performs poorly for detection of *S*. *haematobium* [25], although *S*. *haematobium* is often geographically overlapping and can co-infect individuals in sub-Saharan Africa [26]. Since a sample pooling strategy necessitates a test with high sensitivity, we focused our study in settings endemic with *S*. *mansoni*. Future work can examine incorporation of the circulating anodic antigen (CAA) test or polymerase chain reaction (PCR)-based methods that can detect multiple species of *Schistosoma* to provide a comprehensive strategy [27–31]. Molecular-based (e.g., PCR) techniques are an attractive option for use in pooling strategies because of their high sensitivity (>90%) and specificity, although current methods may be too costly and require advanced laboratory infrastructure that are often out of reach in resource-constrained settings where schistosomiasis is endemic [28–31].

The findings of this investigation should be understood within the limitations of the study design and model assumptions. We simulated theoretical settings by deriving a generalized epidemiologic relationship between prevalence, infection intensity, and parasite dispersion within the population based upon real data [21]. We assumed a negative binomial distribution of disease, which is based upon empiric observation and common practice [21, 22, 32, 33]. To calculate the specificity of the urine pooling strategy, we conservatively assumed that false positives would dilute identically to true positives, although future work should investigate this assumption. While the cost of pooling is uncertain, we assumed each extra pooled sample would incur an additional US$ 0.50 cost and varied this in a sensitivity analysis with our primary findings remaining robust. We defined certainty as the proportion of correct classification ±5% prevalence around the prevalence threshold following common practice, although this threshold for accuracy may be modified. A pooling strategy is best poised to be used in low prevalence settings since this will optimize reductions in number of tests and total cost, but in our study we obtained samples from moderate and high endemicity setting. This was to ensure a sufficient number of positive samples were collected, and to estimate the intrinsic relationship between sensitivity and EPG. We included samples from a wide range of infection intensities, especially light infections. We then modeled a broad range of prevalences (and associated EPG distributions) to capture the epidemiology of a low-endemicity setting. Finally, we did not address the potential for semi-curtailed or curtailed sampling, which is when sampling can be stopped early because of definite classification. However, while this offers an attractive option to reduce testing, often all samples are collected and tested simultaneously making this less practical, although future work could explore this option.

As treatment programs for control and elimination of schistosomiasis are expanded, new tools and strategies are needed to support the efficient targeting of preventive chemotherapy. Further field study of a urine pooling strategy employing POC-CCA cassette test for rapid and convenient *S*. *mansoni* diagnosis is warranted to validate this approach to support control and elimination of schistosomiasis.

## Supporting Information

S1 Text Supplemental materials. (DOCX)

S1 Checklist STARD checklist. (DOCX)
