# Supplementary material for: Evaluation of a Urine Pooling Strategy for the Rapid and Cost-Efficient Prevalence Classification of Schistosomiasis
Source: PLoS Negl Trop Dis. 2016 Aug 9;10(8):e0004894. doi: 10.1371/journal.pntd.0004894 (PMC4978437; doi:10.1371/journal.pntd.0004894)
Supplement: S1 Text — (DOCX) [file pntd.0004894.s001.docx]

**Supplemental Materials**

Lo NC, Coulibaly JT, Bendavid E, N’Goran EK, Utzinger J, Keiser J, Bogoch II, and Andrews JR. Evaluation of a Urine Pooling Strategy for the Rapid and Cost-Efficient Prevalence Classification of Schistosomiasis.

**Contents**

Section 1: Technical appendix.……………..…………….……….……..………………….Page 1

Section 2: Supplemental figures and tables…………………..……………...……….....…Page 13

**Section 1: Technical appendix**

In this supplement, we provide further methodological details relevant to our study. In particular, we include further explanation on the latent class analysis, epidemiological model for *Schistosoma mansoni*, and model assumptions.

*Latent class analysis*

We used a Bayesian latent class analysis (LCA) to estimate the sensitivity and specificity of quadruple Kato-Katz and single circulating cathodic antigen (POC-CCA) test.^1^ This statistical analysis combines prior knowledge on sensitivity and specificity with new observed data to update the estimate on these diagnostic parameters. Most importantly, the LCA simultaneously computes sensitivity and specificity of multiple diagnostic tests without assuming any is the gold standard. This is particularly important for diagnosis of helminthiases, which have no reference standard test with perfect sensitivity and specificity. We used LCA to provide point estimates with associated 95% credible intervals (CI) for sensitivity and specificity of quadruple Kato-Katz and single POC-CCA. The prior knowledge for each test’s sensitivity and specificity required an estimated minimum, mean, and maximum (See Table A1).^2-5^ We used a beta distribution for each parameter. The beta coefficients ($\alpha,\beta)$ are calculated following the equations below.

$$u=\frac{\alpha}{\alpha+\beta} \left( 1 \right)$$

$$\frac{1}{4}*R=\sqrt{\frac{\alpha\beta}{\left( \alpha+\beta\right)^{2}\left( \alpha+\beta+1 \right)}} (2)$$

$$Where:$$

$u$= mean

$R$= total range (max – min)

$\alpha$= alpha coefficient

$\beta$= beta coefficient

In the LCA simulation, we used the Gibbs sampler algorithm (Markov chain Monte Carlo algorithm).^1^ We ran 10,000 simulations and assessed convergence. We tested alternative prior distributions to ensure our findings were robust. The Kato-Katz and POC-CCA were assumed to be independent from one another except on disease status. This assumption is common practice, although there is likely some conditional dependence between these tests. The LCA model parameters (Table A1) and observed data from the study (Table A2) are provided.

*Epidemiological model for schistosomiasis*

The disease distribution for *S. mansoni* was modeled using the negative binomial statistical distribution following observed empirical data and common modeling practice. The negative binomial distribution accounts for the significant overdispersion in worm burden (defined as when variance is greater than the mean). This is because a majority of individuals are uninfected or lightly infected, and only a minority experience moderate or heavy infections. This relationship involves the prevalence (P), mean worm burden (M), and dispersion parameter (k) as shown in equation 3. Importantly, mean worm burden (infection intensity) is often represented as eggs per gram of feces (EPG), which is an indirect measure of mean infection intensity.

$$P=1-\left( 1+\frac{M}{k} \right)^{-k} (3)$$

We developed a generalized relationship for helminth epidemiology based on the approach of prior studies. We performed a literature review in PubMed for relevant articles published in English between January 1, 2010 and March 1, 2016, using the search terms: “*Schistosoma mansoni*” and “prevalence” and “intensity” restricted to the title and abstract field. We included studies in treatment naïve settings that reported the arithmetic mean EPG and fit within the negative binomial distribution. We also included studies from an earlier exploratory analysis that fit this criteria. Ultimately, 19 studies (N=126 data points) were included for *S. mansoni*. We used a 2nd order polynomial (quadratic) relationship to derive the relationship between prevalence and arithmetic mean EPG in infected individuals. This relationship was extended to calculate the dispersion parameter. We used the 95% prediction interval around this relationship to model setting-specific epidemiologic differences, where settings with the same prevalence can have different EPG distributions. Full details are previously described in supplemental methods.^6^

*Additional analytical details and model assumptions*

The sensitivity of the urine pooling strategies was modeled as a logistic regression. The independent variable (arithmetic mean EPG) was used to predict sensitivity of a specified pooling strategy (based on pooling size; N=4, 8, or 12). This analysis used the primary data collected in the study, where one positive urine (positive by Kato-Katz and single POC-CCA) was diluted with three (N=4), seven (N=7), or eleven (N=12) negative urines (see Methods). However, in practical implementation, multiple urine samples may be positive in the same pool. To estimate sensitivity in these cases, we used the total EPG of all positive urines to predict sensitivity (Figure A1). Importantly, this is a conservative assumption that may underestimate sensitivity when multiple positive urines are pooled. This can be understood when pooling strategies (N=4, 8, 12) are normalized under this assumption and then compared to single POC-CCA. This was done through re-assignment of the sensitivity at any given EPG to that EPG divided by the pool size (Figure A2). This resulted in a substantial left shift of the sensitivity curve. We found that pooled sensitivity was underestimated when compared to single POC-CCA (Figure A2), hence making our assumption conservative. Since we modeled low burden settings (prevalence 0-20%), this further relaxes concerns on this assumption since multiple positive samples would be less common. Future work can validate this finding.

We modeled specificity of pooling urine samples using the estimated imperfect specificity of POC-CCA and knowledge of an individual’s “true” infection status (from simulated egg counts). We considered two opposing forces: 1) pooling of urine increases the chance that a false positive urine would be included; and 2) urine pooling decreases the probability of detecting a false positive through a “dilution effect” from addition of multiple negative urines. We modeled specificity through the equations below and a binomial distribution, using parameters gathered from primary data (see Table 2, Table A3).

$$Sp_{D4}=\Pr\left( 0 \right)|{Sp}_{cca}+(Pr\left( 1 \right)|{Sp}_{cca})*{Dil}_{4}$$

$$Sp_{D8}=\Pr\left( 0 \right)|{Sp}_{cca}+(Pr\left( 1 \right)|{Sp}_{cca})*{Dil}_{8}+(Pr\left( 2 \right)|{Sp}_{cca})*{Dil}_{4}$$

$$Sp_{D12}=\Pr\left( 0 \right)|{Sp}_{cca}+(Pr\left( 1 \right)|{Sp}_{cca})*{Dil}_{12}+(Pr\left( 2 \right)|{Sp}_{cca})*{Dil}_{8}+(Pr\left( 3 \right)|{Sp}_{cca})*{Dil}_{4}$$

$$Where:$$

$${Sp}_{DN}=specificity of pooling strategy; pool size of N$$

$$\Pr\left( N \right)=probability of N positive urines in pooled sample (based on binomial dist)$$

$${Sp}_{cca}=specificity of POC-CCA test$$

$${Dil}_{N}=dilution effect, pool size of N$$

Specificity was also computed by pooling negative urines in pools of 4, 8, and 12 (see Table 3 and Methods).

We calculated the sample size necessary to estimate sensitivity of the urine pooling strategies, treating the POC-CCA as a binary test.^7^ Since all pooled urines samples were positive (see Methods), the prevalence was 100%. We aimed to achieve a 95% confidence level with 10% maximum marginal error of estimate. Based on literature on the POC-CCA test, we estimated a sensitivity of 80% (see Table A1). The computed sample size from these assumptions was 62. We used a final sample size of N=78 for the urine pooling study, which was within the estimated required sample size.

We used Python to develop the microsimulation and latent class analysis, and R for regression analysis; both programs were used for data visualization (including the R package, ggplot2).

*Quality control in field and laboratory procedure*

We randomly selected 10% of Kato-Katz thick smears for quality control, including both positive and negative slides. These slides were re-examined by a senior technician and considered correct if the following tolerance margin was not exceeded: 1) No difference in presence/absence of *S. mansoni*; 2) Egg counts were +/-10 eggs for counts ≤100 eggs or +/-20% for counts >100 eggs. If the tolerance margin were exceeded in one or more slide, all slides were re-read by the technicians. The new results were discussed to reach consensus.

No adverse events were noted during the sample procurement and diagnostic testing.

**Table A1**: **Model parameters for latent class analysis**

|  |  | Kato-Katz (quadruple) | | |  |  | POC-CCA (single) | | |
| --- | --- | --- | --- | --- | --- | --- | --- | --- | --- |
|  |  | | Beta coefficient | | |  | | Beta coefficient | |
|  | Range (%) | | α | β | | Range (%) | | α | β |
| Sensitivity | 60-80 | | 58.1 | 24.9 | | tr(+) 80-100  tr(-) 50-90 | | 31.5  14 | 3.5  6 |
| Specificity | 90-100 | | 71.3 | 3.8 | | tr(+) 60-90  tr(-) 80-100 | | 24.3  31.5 | 8.1  3.5 |

Ranges for sensitivity and specificity were based upon literature.^2-5^

tr; POC-CCA trace test result, treated as positive (+) or negative (-)

**Table A2: Study observations for latent class analysis**

|  | | KK+, CCA+ | KK-, CCA+ | KK+, CCA- | KK-, CCA- |
| --- | --- | --- | --- | --- | --- |
| Trace positive | |  |  |  |  |
|  | Overall | 66 | 13 | 2 | 33 |
|  | Light intensity | 40 | 13 | 2 | 33 |
|  | Moderate intensity | 13 | 13 | 0 | 33 |
|  | Heavy intensity | 13 | 13 | 0 | 33 |
| Trace negative | |  |  |  |  |
|  | Overall | 52 | 5 | 16 | 41 |
|  | Light intensity | 26 | 5 | 16 | 41 |
|  | Moderate intensity | 13 | 5 | 0 | 41 |
|  | Heavy intensity | 13 | 5 | 0 | 41 |

KK; quadruple Kato-Katz, CCA; POC-CCA test

**Table A3: Dilution effect of true positives with the**

**urine pooling strategy**

|  | Sensitivity | | | |
| --- | --- | --- | --- | --- |
| Pool size |  | **Logistic model^a^** | **Primary data^b^** |  |
| 4 | 0.23 | | 0.21 | |
| 8 | 0.10 | | 0.07 | |
| 12 | 0.01 | | 0.00 | |

^a^Sensitivity computed from logistic model; dilution effect on POC-CCA score of 1+ (see Figure A3).

^b^Sensitivity computed from empirical data; dilution effect on low intensity infections (EPG<20)

POC-CCA trace results were treated as negative in this analysis.


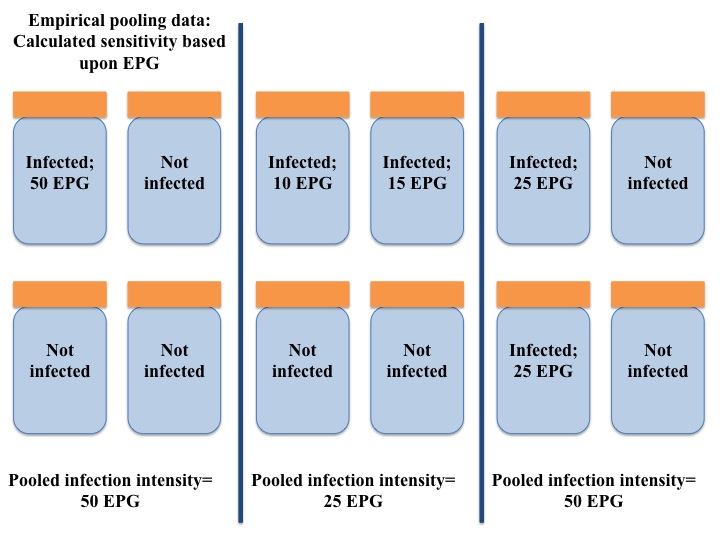


**Figure A1: Calculation of eggs per gram for pooled urine sample.** The pooled infection intensity was computed as the summation of the EPG from all positive urine samples. EPG; eggs per gram of feces.


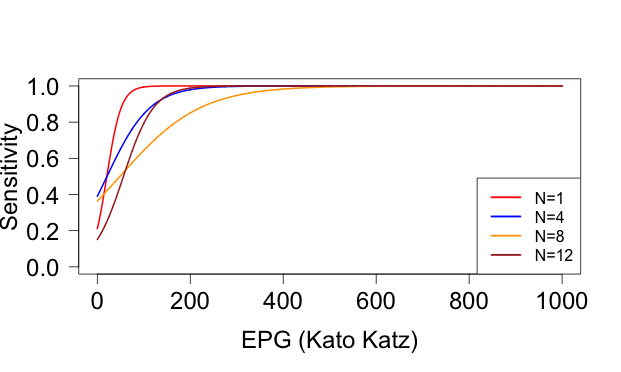


**Figure A2: Normalization of the sensitivity of pooled urine strategies to single POC-CCA.** The pooled urine samples were adjusted to assign their sensitivity at a given EPG to the EPG divided by their pool size.


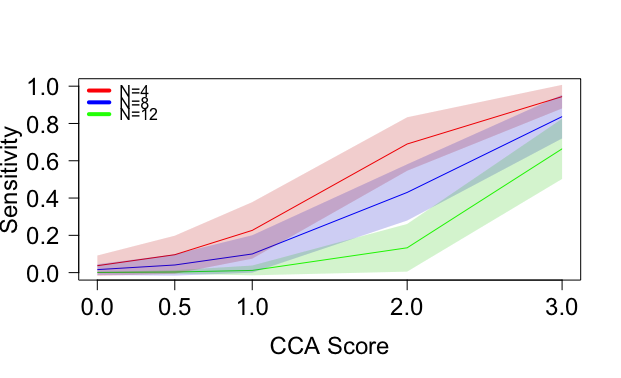


**Figure A3: Effect of sample dilution on sensitivity of POC-CCA test.** The dilution effect was modeled with a logistic regression, where the binary dependent variable was detection of the positive urine after dilution (POC-CCA trace result as negative) and the predictor (independent) variable was POC-CCA score (trace-0.5, 1+, 2+, 3+). The dilution effect was estimated from an initial POC-CCA score of 1.0 (see Table A3).

**References**

1. Joseph L, Gyorkos TW, Coupal L. Bayesian estimation of disease prevalence and the parameters of diagnostic tests in the absence of a gold standard. *Am J Epidemiol* 1995; **141**(3): 263-72.

2. Coulibaly JT, N'Gbesso YK, Knopp S, et al. Accuracy of urine circulating cathodic antigen test for the diagnosis of Schistosoma mansoni in preschool-aged children before and after treatment. *PLoS Negl Trop Dis* 2013; **7**(3): e2109.

3. Lamberton PH, Kabatereine NB, Oguttu DW, Fenwick A, Webster JP. Sensitivity and specificity of multiple Kato-Katz thick smears and a circulating cathodic antigen test for Schistosoma mansoni diagnosis pre- and post-repeated-praziquantel treatment. *PLoS Negl Trop Dis* 2014; **8**(9): e3139.

4. Shane HL, Verani JR, Abudho B, et al. Evaluation of urine CCA assays for detection of Schistosoma mansoni infection in Western Kenya. *PLoS Negl Trop Dis* 2011; **5**(1): e951.

5. Silveira AM, Costa EG, Ray D, et al. Evaluation of the CCA Immuno-Chromatographic Test to Diagnose Schistosoma mansoni in Minas Gerais State, Brazil. *PLoS Negl Trop Dis* 2016; **10**(1): e0004357.

6. Lo NC, Lai YS, Karagiannis-Voules DA, et al. Assessment of global guidelines for preventive chemotherapy against schistosomiasis and soil-transmitted helminthiasis: a cost-effectiveness modelling study. *The Lancet Infectious diseases* 2016.

7. Hajian-Tilaki K. Sample size estimation in diagnostic test studies of biomedical informatics. *J Biomed Inform* 2014; **48**: 193-204.

8. Worrell CM, Bartoces M, Karanja DM, et al. Cost analysis of tests for the detection of Schistosoma mansoni infection in children in western Kenya. *Am J Trop Med Hyg* 2015; **92**(6): 1233-9.

**Supplemental figures and tables**

Table S1: Estimated costs for single and pooled diagnostic tests

Table S2: Estimated prevalence of schistosomiasis from latent class analysis

Table S3: Decision rule for LQAS with various numbers of tests for Kato-Katz, single POC-CCA, and urine pooling strategy

Figure S1: STARD-flowchart showing study participation

Figure S2: Relationship between prevalence and propotion positivity for single and pooled diagnostic test for schistosomaisis with perfect diagnostic tests.

Figure S3: Relationship between prevalence and percent positivity for single and pooled diagnostic test for schistosomaisis with imperfect diagnostic tests

Figure S4: Modeled estimation of the sensitivity of the urine pooling strategy using POC-CCA trace result as positive

Figure S5: Number of tests and total costs per each correctly classified school from microsimulation analysis of urine pooling strategy and traditional stool microscopy

Figure S6: Operating Characteristic curves for microsimulation analysis using POC-CCA trace result as positive

Figure S7: Number of tests and total costs from microsimulation analysis using POC-CCA trace result as positive

Figure S8: One-way sensitivity analysis of microsimulation using POC-CCA trace result as positive

Figure S9: Operating Characteristic curves for microsimulation analysis using a 5% prevalence threshold

**Table S1: Estimated costs for single and pooled diagnostic tests**

|  | Supplies (US$) | Labor (US$) | Pooling (US$) | Total cost (US$) |
| --- | --- | --- | --- | --- |
| Kato-Katz (single) | 1.09 | 2.9 | 0 | 3.99 |
| POC-CCA test | 3.15 | 1.98 | 0 | 5.13 |
| Pooled POC-CCA (N=4) | 3.15 | 1.98 | 1.5 | 6.63 |
| Pooled POC-CCA (N=8) | 3.15 | 1.98 | 3.5 | 8.63 |
| Pooled POC-CCA (N=12) | 3.15 | 1.98 | 5.5 | 10.63 |

Data includes cost for supplies and labor only, with an estimated US0.50 per additional pooled urine sample.

Data obtained from literature.^8^

**Table S2: Estimated prevalence of schistosomiasis from**

**latent class analysis**

| Diagnostic tests | Prevalence (95% CI) |
| --- | --- |
| Kato-Katz +  POC-CCA (trace as positive) | 67.7 (57.5, 76.9) |
| Kato-Katz +  POC-CCA (trace as negative) | 67.7 (57.5, 76.9) |

**Table S3: Decision rule for LQAS with various numbers of tests**

**for Kato-Katz, single POC-CCA, and urine pooling strategy**

|  | Number of tests | | |
| --- | --- | --- | --- |
|  | **N=20** | **N=50** | **N=250** |
| Kato-Katz (single) | 3 | 6 | 29 |
| POC-CCA test | 3 | 6 | 31 |
| Pooled POC-CCA (N=4) | 4 | 10 | 50 |
| Pooled POC-CCA (N=8) | 5 | 12 | 61 |
| Pooled POC-CCA (N=12) | 3 | 7 | 36 |

The decision rule refers to the number of positive tests which results in a batch

being considered positive (above 10% prevalence threshold) for LQAS.

**Figure S1: STARD-flowchart showing study participation**

| **a** | **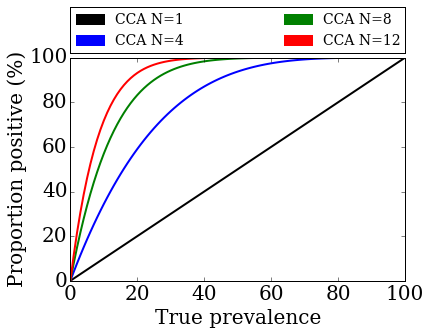** | **b** | **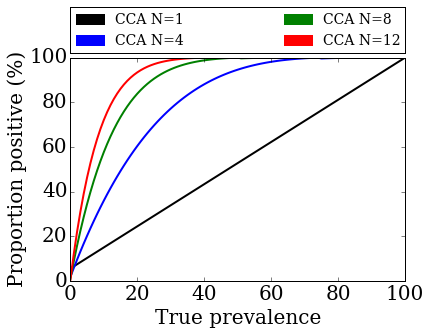** |
| --- | --- | --- | --- |
|  | **Figure S2: Relationship between prevalence and propotion positivity for single and pooled diagnostic test for schistosomaisis with perfect diagnostic tests.** The microsimulation from the study was tested with (a) perfect sensitiivty and specificity; and (b) perfect sensitivity and imperfect specificity. | | |


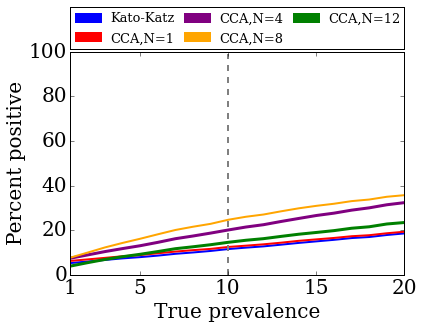


**Figure S3: Relationship between prevalence and percent positivity**

**for single and pooled diagnostic test for schistosomaisis with**

**imperfect diagnostic tests**

**
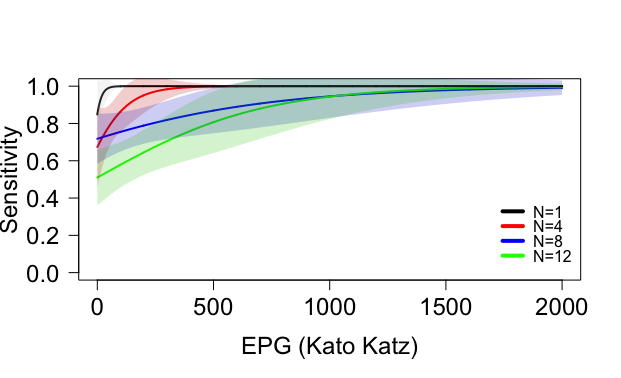
**

**Figure S4: Modeled estimation of the sensitivity of the urine pooling strategy using POC-CCA trace result as positive**

| **a** | **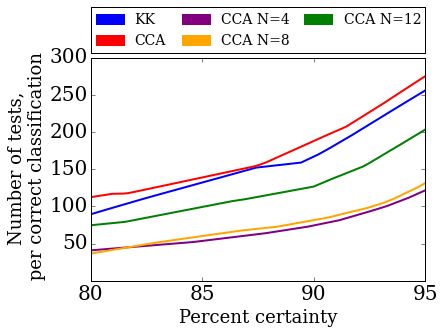** | **b** | **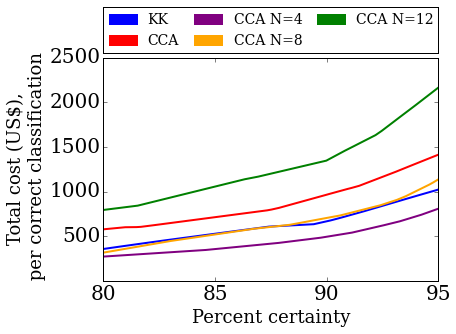** |
| --- | --- | --- | --- |

**Figure S5: Number of tests and total costs per each correctly classified school from microsimulation analysis of urine pooling strategy and traditional stool microscopy.**

| **a** | **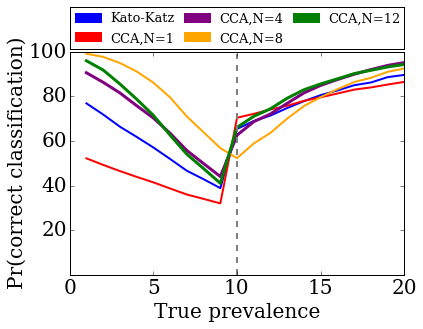** | **b** | **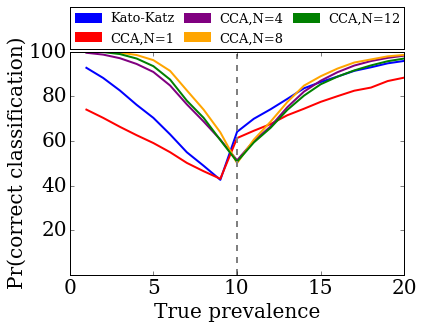** |
| --- | --- | --- | --- |
| **e** | **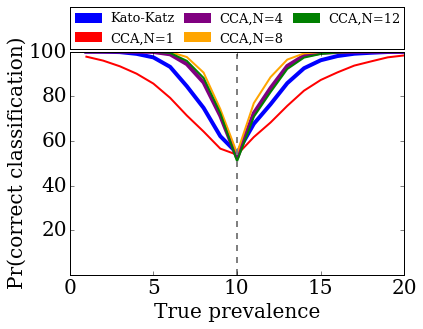** | **d** |  |

**Figure S6: Operating Characteristic curves for microsimulation analysis using POC-CCA trace result as positive.** Using primary data on urine pooling strategies (N=4, 8, 12 samples), we used a microsimulation to model the probability of correct binary classification around a prevalence threshold to indicate need for school-based preventive chemotherapy according to WHO (10% prevalence). The results are presented for: a) 20 tests; b) 50 tests; and c) 250 tests. We compared traditional stool microscopy (duplicate Kato-Katz, one stool sample), single POC-CCA test, and the three urine pooling strategies (N=4, 8, 12 pool) using the WHO 10% prevalence threshold. POC-CCA trace results are treated as positive.

| **a** | **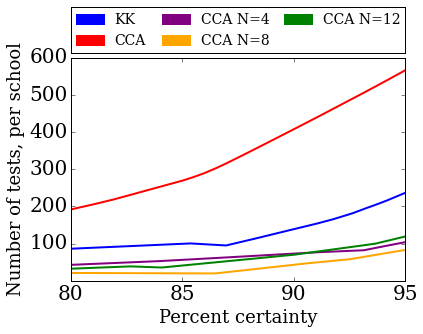** | **b** | **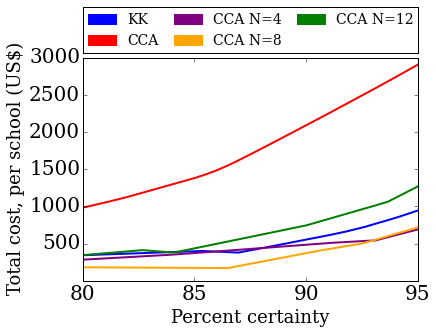** |
| --- | --- | --- | --- |

**Figure S7: Number of tests and total costs from microsimulation analysis using POC-CCA trace result as positive.** The urine pooling strategy (N=4, 8, 12 pool) was compared against stool microscopy to estimate the number of tests (a) and total cost (b) for identical level of certainty in binary classification on need for preventive chemotherapy. POC-CCA trace results were treated as positive. We used a 10% prevalence threshold in this base case analysis. A loess algorithm was applied for visualization.

| **a** |  |
| --- | --- |
| **b** |  |

**Figure S8: One-way sensitivity analysis of microsimulation using POC-CCA trace result as positive.** This analysis tested the effect of changing individual model parameters on the total cost of the urine pooling strategy for a) pool of four (N=4); b) pool of eight (N=8). POC-CCA trace results were treated as positive. The horizontal bar represents the total cost to achieve 90% level of certainty (+/-5% around the prevalence threshold) in classification across a range of values for the tested parameter. The y-axis (solid black line) represents the total cost of the urine pooling strategy under base case assumptions. All strategies to the left of US$555 indicate a cost saving advantage compared to traditional stool microscopy under base case assumptions.

| **a** | **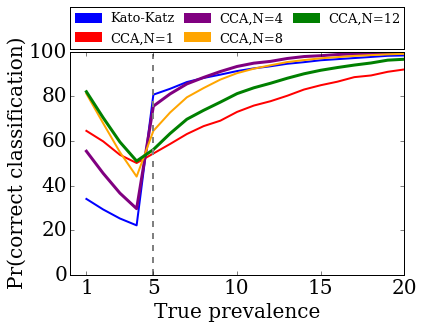** | **b** | **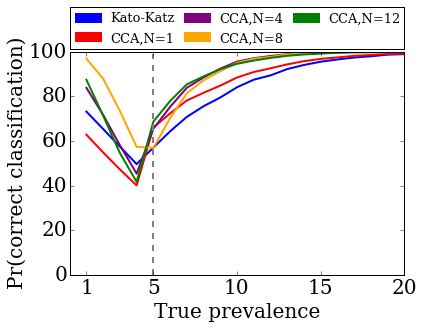** |
| --- | --- | --- | --- |
| **e** | **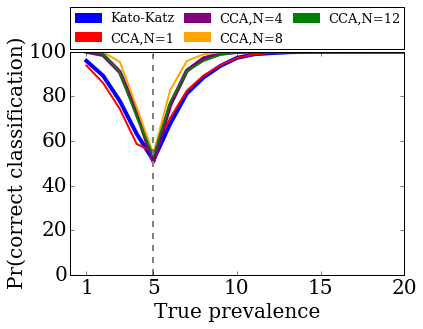** | **d** |  |

**Figure S9: Operating Characteristic curves for microsimulation analysis using a 5% prevalence threshold**
